# Inducing perceived group variability triggers the incorporation of counter-stereotypic information into a generalized stereotype change

**DOI:** 10.1038/s41598-024-59929-0

**Published:** 2024-04-22

**Authors:** Ana Sofia Santos, L. Garcia-Marques, T. A. Palma, J. Reese

**Affiliations:** 1 Affiliation, Faculdade de Psicologia, Universidade de Lisboa, Alameda da Universidade, 1649-013 Lisbon, Portugal; 2 CICPSI, Faculdade de Psicologia, Universidade de Lisboa, Alameda da Universidade, 1649-013 Lisbon, Portugal

**Keywords:** Human behaviour, Psychology

## Abstract

Perceived variability is the extent to which individuals perceive group members as being similar to one another. Previous research has focused on how: group variability is perceived (and measured); information indicative of group heterogeneity can lead to reductions in stereotypicality; or how stereotype-inconsistent information can result into increased perceived variability. The present combines the three lines of research into a single research venue. In previous studies the stereotypicality of a group representation was influenced by priming stereotype-unrelated traits in an unrelated-context, prior to stereotype measurement; but priming counter-stereotypic traits had no effect on stereotypicality, although it boosted perceptions of group's variability. The present study examines whether highlighting dissimilarities among members of the same professional groups results in subsequent changes in the reported stereotype for a, not yet mentioned, group. The more the dissimilarity among group members, the more likely individuals were to incorporate counter-stereotypic information into the targeted-group, described as less stereotypic, even in central tendency measures. Importantly, the generating mechanism may involve a modification of participants' overall perception of variability. When members within professional groups are perceived as dissimilar, the well-known resistance of stereotypes to counter-stereotypic information is lessened making the group representations more flexible and less biased.

## Introduction

Stereotype persistence is a well-researched phenomenon that can be characterized by the paucity of stereotype revision in response to diagnostic counter-stereotypic information (for reviews, see^1,2^). Past literature has attempted to unearth clearer insights into the specific circumstances under which inconsistent information prompts stereotype revision—whether efficiently or not^2–12^. At first, the word “change” within literature regarding stereotype change concerned change in the modal values (or central tendencies) of corresponding group representation, but soon it became obvious that there were other dimensions of group representations that were worth of consideration, namely, perceived variability. Perceived variability differed in judgments relative to in- and outgroups and thus, a whole literature about perceived variability and the best ways to measure it developed in eighties and nineties^13,14^. More recently, several researchers have argued that increased perceived group variability could lead to stereotype change in perceived central tendencies. And conversely, that stereotype-incongruency could lead to increased perceived variability. However, the full picture of the role of stereotype-incongruency and perceived group variability has yet to be drawn.

Most of the previous attempts to change stereotypes have provided information about group members who are deviant in various ways or to varying degrees^5,8,11,15^, but have only tested the impact of disconfirming information presented (either dispersed across several group members or concentrated in a small number of members) on central tendency as a dependent measures^2,3,5–7,9–11,15–21^. Rarely have they addressed whether or not disconfirming information triggered changes in perceived group variability as a dependent measure of generalized change.

Fewer studies have directly demonstrated that stereotype change can extend beyond mere central tendency revision^8,12,23–27^, stressing that changes in perceived group variability can also manifest as a result of such shifts, thus, bringing attention to the fact that perceived variability estimates are adaptable parameters (for similar results in prejudice and discrimination, see^22^). Specifically, they were able to elevate perceived group variability dependent measures through the introduction of inconsistent members tailored to a specific target group^8,23–27^. With this, they were capitalizing two literatures and specifying how stereotype change and manipulation of perceived group variability could be complementary^27^. Indeed, results suggested that participants exposed to incongruent information tended to select more variable distributions as more reflective of the entire group. Participants evaluated the group as having wider ranges on relevant dimensions and formed more differentiated distributions of group members along stereotyped trait dimensions compared to other participants^5,8,23,25–27^. When the manipulation involved was the stereotype-inconsistent information being heterogeneous (inconsistent exemplars differ among themselves) or homogeneous (the inconsistent exemplars are alike)^24^, the heterogeneous condition led to significant stereotypes changes (particularly in perceived variability measures), when compared to the homogeneous condition.

Parallel results were observed in studies where counter-stereotypic traits were contextually primed through an unrelated task and subsequent assessments were made to determine whether those primed concepts were incorporated into the later reported stereotype for the group as a whole^28^. Results suggested that contextually activated counter-stereotypic information, in comparison to stereotype-unrelated information, was less likely to be accepted as representative of one's stereotypic beliefs, and had no impact on trait selection, typicality ratings, or perceived central tendency measures. Nevertheless, it does lead participants to perceive the target group as more variable^28^. Those studies about the effect of priming on perceived group variability were able to bring insight from the stereotype change literature by drawing attention to key variables like perceived variability, which were not yet considered by priming literature. Nevertheless, further research must yet tapped into the challenge of if, and how long, these stereotype reduction effects caused by counter-stereotypic information are sustained.

The present study attempts to combine three lines of research: (i) stereotype-incongruent information in the perceived central tendency of stereotyped groups, (ii) the impact of stereotype-incongruent information in perceived variability, and (iii) the effects of perceived variability in stereotype change. The objective of the current study is to investigate whether a one-time experimental manipulation designed to change perceived group variability through the increased awareness that within-group members of same professional groups are diverse and varied, results in subsequent changes to the reported stereotype for the targeted professional group, namely, by overcoming the well-known resistance of stereotypes to contextually primed counter-stereotypic information ^28^. The present study will assess these changes in stereotype generalization through perceptions of the groups’ central tendencies (typical characteristics) and perceived group variability.

## Perceived variability and stereotyping

Social groups vary in how they are perceived, either as having members that greatly differ or that are very similar with each other, regarding a given trait^13,14,29–31^. For certain social groups, individuals may perceive members as dissimilar while, for others, members are seen as resembling to each other^32^. The term “perceived variability” refers to the extent to which individuals see a group as either heterogeneous or homogenous, with higher perceived variability considered a desirable outcome ^13,14,22,33–39^. Most of the empirical studies published on the topic stressed the specific determinants of perceived group variability (see^40–43^, for reviews), but, as mentioned before, some authors enhanced also its important implications for stereotype change^8,25,26,44^. As noted by^44^, rather than persuading individuals to abandon their beliefs about group characteristics, a potentially more successful strategy can involve fostering an appreciation for the diversity among group members regarding those characteristics. One of the ways to increase individuals’ perception of variability was to increase awareness of distinct subgroups that exist within a unified outgroup^22,42,45–47^; (for an analogous approach, see^48^). Recent demographic trends have been promoting working groups exhibiting greater variety^33,34^; and we can speculate such heterogeneity in within-group members may consubstantiate in increasingly diverse societies, naturally reducing perceived differences between groups, and making differentiated stereotypes less visible^22,35^.

In theory, central tendency and perceived variability represent distinct parameters of the mental representation of social groups. The former assesses the extent to which group members, on average, exhibit a specific trait. If a particular trait is perceived as greatly representative of group members (e.g., viewing librarians as introverted) a higher perceived central tendency is associated to that trait. On the other hand, variability pertains to the notion that not all group members are alike ^23,35,43^, meaning that not all group members share the trait in the same extent. Beyond the simplification that perceived variability is the acknowledgment that everyone is different, its mental representation suggests a nuanced concept, able to enlighten the interplay between social categories and traits^2,14,32,39,44,47,49^.

Such connection between perceived variability and stereotyping has been substantiated experimentally ^24,25,43,44,50,51^, and incentivized some authors to use these terms interchangeably^13,45^, suggesting that the association between both constructs may arise from the fact that stereotyping is, in essence, a mathematical outcome of perceived variability^22,35^. When individuals perceive that not all group members are alike in a specific characteristic, they are less inclined to associate that characteristic with the entire group, bringing a more complex representation of the target group ^52^, and becoming less likely to associate characteristics in an all-or-nothing manner. Maintaining a fixed, generalized stereotype toward an entire group becomes challenging when convinced that group members are dissimilar. Further, a highly variable stereotype provides less informative content^35^. A study by^50^ found that individuals exhibit more confidence in judgments made after applying a low-variability (homogeneous) stereotype compared to a high-variability (heterogeneous) stereotype^51^. Also, individuals are quicker to determine whether an attribute accurately describes a group when the previously encountered information about that trait is relatively invariable, compared to when is highly variable^53^. The current study goes back to the effects of construct priming on stereotype assembling^28^ and extends it to the role of anticipatedly manipulating perceived group variability, before priming a counter-stereotypic construct.

## Effects of construct priming on stereotype assembling

Past studies have supported the idea that stereotypes are flexible and adaptable constructs, with components that can be rearranged into a ‘working stereotype’, when necessary^28,54,55^. Across three experiments^28^, constructs with varying degrees of stereotype relevance were activated in an unrelated context to examine whether these concepts influenced the content or variability of subsequent assessed stereotypes. An unrelated linguistic task was used to activate concepts immediately before assessing stereotypes. The effects of priming counter-stereotypic or stereotype-unrelated traits on a subtler measure of stereotype fluidity (i.e., the perceived dispersion of members over corresponding trait dimensions) were explored. The findings indicated that, when compared to stereotype-unrelated information, contextually activated counter-stereotypic information is less prone to being acknowledged as representative of one's stereotypical beliefs. Moreover, it does not influence trait selection, typicality ratings, or perceived central tendency measures. However, it does result in participants viewing the target group as more diverse.

These effects were argued to align with models that emphasize applicability‐driven priming effects, such as the Synapse Model^56–58^, which posits the activation of a construct reflects its relevance in a given context^59^. Subtle priming of specific contexts or exemplars tends to activate prime-consistent stereotypic content ^56,60^, however, the applicability of an activated construct may be constrained by the relative applicability of other available constructs^57,58^. Thus, while accessible stereotype-unrelated information may more easily be incorporated into the assessed beliefs and be applied, it is less likely that contrasting activated concepts, inconsistent with the majority of activated stereotypic beliefs, would be readily incorporated. Nevertheless, its presence may still be reflected in measures of perceived variability, as the simultaneous activation of opposing traits suggests higher perceived group variability^28^. Those findings are also consistent with the Situated Inference Model^61^, which explains individuals interpret primed information based on their immediate situation^62,63^ (for a taxonomy of experimental procedures addressing priming effects, see^64^).

A key point of those results is that, unlike typical stereotype change or stereotype priming procedures (as reviewed by ^5,45^), the primed information was never linked to group members or the group as a whole. This particularity of the paradigm is essential as it guarantees primed information was not deliberatively used to revise the stereotype.

The following study investigated the relationship between manipulating perceived variability, overall, and stereotype change, in particular, for a target group, measuring stereotype change through the likelihood of incorporating a subtly contextually primed counter-stereotypic information into the stereotype reported for a specific (i.e., not previously involved) target group presented afterward. We posit that initial exposure to a cover story that uses a supposed scientific report about professional groups to convey information emphasizing the heterogeneity of members within the same professional groups, as opposed to evidence supporting their homogeneity, fosters an awareness of the potential dissimilarity among individuals sharing occupations, specifically when primed by counter-stereotypic information. Consequently, this heightened awareness increases, when facing a contextually primed information that is counter-stereotypic in nature, the likelihood of incorporating it into the reported stereotype for the targeted professional group.

## Experiment

The current study leverages assumptions from Situated Inference Model^61^ to underscore the role perceived group variability manipulation can play in stereotype change, facilitating the overcoming of stereotype resistance to contextually primed counter-stereotypic information.

In the subsequent experiment, we followed the methodology outlined by^28^, contrasting conditions where individuals' attention was directed towards either the overall variety (heterogeneous condition) or the overall similarity (homogeneous condition) of within-group members of same professional groups. The aim was to investigate whether participants exposed to plausible heterogeneous information would modify their mental representation about a specific targeted group by incorporating the contextually primed counter-stereotypic information immediately before reporting the stereotype. In contrast to previous studies with the same paradigm^28^, this was expected to influence the group's central tendencies and typical characteristics in addition to the perceived group variability measures. Simultaneously, we anticipated that, when exposed to plausible homogeneous information, these effects would dissipate across all parameters of the mental representation used to assess stereotype change.

## Methods

### Participants and design

A total of 165 students from the University of Lisbon willingly participated in the study upon the researcher’s request. After excluding five responses from non-Portuguese natives, a total of 160 responses were recorded, consisting of 137 females and 23 males, with ages ranging from 18 to 49 (*M* = 19.87, *SD* = 4.08). Participants were randomly assigned to a 2 variability scenario (homogeneous or heterogeneous) × 2 prime (intelligence or friendliness) × 2 target group (construction worker or disco bouncer) × 2 trait type (stereotype-unrelated and counter-stereotypic) mixed factorial design, with the last factor being within-participants. The sample size was determined by a priori power analysis conducted using G*Power^65^. Past research on this topic^23,28,55^, led us to expect medium-sized effects (based on the conventions established by^71^). We assumed a medium effect size and a correlation of 0.5 between measures. The key hypothesis rests on an interaction between 2 variability scenarios (homogeneous or heterogeneous) and 2 primes (counter-stereotypic or stereotype-unrelated); previous studies used as references commonly observed no differences between the type of occupations and the type of traits used, usually proceeding to the analysis with both variables collapsed. The power analysis showed that 148 subjects would be required for 0.88 power with an alpha level of 0.05. We ran more participants than needed because there was a surplus in the participant pool, and we wanted a buffer against incomplete data. Participants engaged in the study without facing any risks. Ethical approval was obtained from the Ethics Committee of the Faculty of Psychology, University of Lisbon. The research adhered to the code of the American Psychological Association (2012, 2018), and all methods were conducted in accordance with the applicable guidelines and recommended regulations.

### Material

#### Manipulation of perceived variability

In prior studies, diversity beliefs were manipulated to persuade participants either of the value of diversity or the value of similarity for group activity^36^. In our current research, we modified this procedure by having participants read one of two excerpts resembling a scientific communication text. In the homogeneous condition, participants’ attention was directed towards evidence emphasizing similarities in personal characteristics within members of the same professional, even when comparing members from different social, geographical, and organizational contexts. Contrary to psychological expectations, the heterogeneous condition drew participants’ attention to evidence highlighting differences in personal characteristics within the same professional group members, across those same contexts^36^. For other approaches to manipulating perceived group variability, refer to^8,23,24,27,50–53,66^.

To ensure that the texts conveyed a framework diverging in overall perceived variability, both texts underwent pretesting with participants who did not take part in the main experiment. Ratings from the pretest revealed that the homogeneous condition led to significantly lower perceived variability ratings than the heterogeneous condition.

#### Pretest of the texts used to manipulate perceived variability attributed within members of same professional groups

A total of 40 participants read one of two texts and rated the extent to which the text clearly communicated the message, how well ideas were articulated, and the quality of information transmitted in a 9-point rating scale ranging from “not at all” to “totally”. Additionally, participants rated perceived variability through a one-item question in which indicated the extent to which they thought members of the same professional groups were different from each other (on a continuous rating scale with endpoints labeled “not at all different” and “very different”).

Independent samples t-test revealed that the text in the homogeneous condition conveyed an image that was just as clear (*M* = 7.60, *SD* = 1.35) as the text in the heterogeneous condition (*M* = 7.85, *SD* = 0.67), *t*(38) = -0.74, *p* = 0.464, *d* = -0.23; transmitted ideas as clearly (*M* = 7.00, *SD* = 1.26) as the text in the heterogeneous condition (*M* = 6.95, *SD* = 1.54), *t*(38) = 0.11, *p* = 0.911, *d* = 0.04; and were as informative (*M* = 7.80, *SD* = 0.89) as the text in the heterogeneous condition (*M* = 7.85, *SD* = 0.75), *t*(38) = -0.19, *p* = 0.849, *d* = -0.06. Importantly, an independent samples t-test revealed that the text in the homogeneous condition led to significant lower perceived variability ratings (*M* = 3.10, *SD* = 1.02) than the text in the heterogeneous/variable condition (*M* = 6.20, *SD* = 1.20), *t*(38) = − 8.82, *p* < 0.001, *d* = − 2.79.

#### Choice of group counter-stereotypic and stereotype-unrelated traits

The selection of professional groups and primed concepts was guided by earlier work outlined in^23,24,28,55^, and for a detailed procedure, refer to^23,24^. Indeed, previous studies^23,24,28^ (after pretesting) identified four target groups with strong consensual stereotypes: computer programmers (intelligent), construction workers (not intelligent), childcare professionals (friendly), and disco bouncers (unfriendly). We chose two of these target occupational groups because they were consensually identified as strong (i.e., clear-cut) stereotypes in contemporary Portuguese society and thus provided a conservative test for our counter-stereotype priming permeability hypothesis. Traits were classified as counter-stereotypic for a group if they were never chosen as the best descriptor of that group and simultaneously were the antonym of the best descriptor, by more than 20% of pretested participants. Traits were considered stereotype-unrelated if neither the trait nor its antonym was ever chosen as the best descriptor of a group. Following these criteria and pretests conducted by^28^, we identified construction workers as described as unintelligent and bouncers as described as unfriendly. Consequently, by priming intelligence or friendliness, we aimed to prime the counter-stereotypic traits of construction workers and bouncers, respectively^23^. Additionally, we primed stereotype-unrelated traits, where intelligence and non-intelligence were stereotype-unrelated for bouncers, and friendliness and unfriendliness were stereotype-unrelated for construction workers.

### Procedure

Participants underwent testing in small group sessions, with a maximum of 10 individuals. Upon arrival, participants were seated in front of computers in individual workstations separated by barriers. All instructions and stimuli were presented on the computer, individually, to prevent participants from engaging in group dynamics that might influence their responses. Participants were informed that they would be partaking in a series of unrelated studies: an initial study assessing the comprehensibility of reports oriented towards scientific communication, a linguistics study, and an investigation on impressions of social groups. To enhance the illusion of three separate experiments, instructions and questions for each task were presented using distinct fonts, layouts, and institutional logos.

#### Manipulation of perceived variability

The excerpts from a fictitious scientific communication report served as the cover story to manipulate perceived variability among members of the same professional groups. Participants were randomly allocated to either the heterogeneous or homogeneous condition and were subsequently tasked with evaluating the except on both a comprehensibility rating scale and an informativeness rating scale, to reinforce the cover story’s intended purpose.

#### Trait priming

The linguistic study functioned as a priming manipulation consisting of two components. In accordance with the approach outlined by^28^, participants initially offered familiarity judgments for various words as part of the cover story, and subsequently, participants were tasked with supplying dictionary-like definitions for two words: a neutral trait (i.e., conservative) and a primed trait (i.e., intelligence or friendliness).

#### Trait descriptiveness and perceived dispersion measure

Following the completion of the priming task, participants were informed that the final study, focused on the assessment of stereotypes, aimed to understand how individuals form impressions of social groups. After a general introduction highlighting the significance of research on individuals’ impressions of social groups, participants received task-specific instructions as detailed in^28,54^. Participants proceeded to evaluate the target group using two 9-point trait-rating scales anchored by the trait and its antonym, corresponding to the traits used as primes across conditions (unintelligent vs. intelligent and unfriendly vs. friendly). Subsequently, all participants were presented with an array of 15 distributions systematically combining five levels of central tendency and three levels of dispersion. They were tasked with selecting the distribution they believed best represented the target group as a whole for the stereotypic dimension and again for the stereotype-unrelated dimension^23,24,28^. Funnel debriefing was then employed to assess participants' theories or suspicions regarding any connections between the various tasks provided.

### Ethical approval

The research from which this data was extracted was approved by the Ethics Committee of the Faculty of Psychology, University of Lisbon, in compliance with the code of the American Psychological Association (2012, 2018). This article does not contain any studies with animals performed by any of the authors.

### Informed consent

Informed consent, provided participants with detailed information about the study aims and procedures, and was obtained from all participants. Confidentiality and protection of anonymity were both assured to all the participants.

## Results

As there were no observable differences between the type of occupation condition and the type of traits used, both variables were collapsed and the subsequent analysis categorizes traits used as stereotype-unrelated and counter-stereotypic for both occupation stereotypes included (see Tables 1, 2, and 3).

**Table 1:** Descriptiveness ratings of stereotypic and stereotype-unrelated dimensions across the priming and variability conditions in Experiment 1.

Type of prime	Stereotypic dimension		Stereotype-unrelated dimension	
	Heterogeneous condition	Homogeneous condition	Heterogeneous condition	Homogeneous condition
Counter-stereotypic trait	5.38 (1.19)	8.18 (0.81)	5.33 (1.19)	4.80 (0.79)
Stereotype-unrelated trait	7.38 (0.90)	8.20 (0.85)	6.15 (1.12)	4.75 (1.21)

Numbers outside of the parentheses represent the means, while numbers inside of the parentheses represent the standard deviations.

**Table 2:** Perceived central tendency of stereotypic and stereotype-unrelated dimensions across the priming and variability conditions in Experiment 1.

Type of prime	Stereotypic dimension		Stereotype-unrelated dimension	
	Heterogeneous condition	Homogeneous condition	Heterogeneous condition	Homogeneous condition
Counter-stereotypic trait	2.35 (1.03)	4.20 (0.82)	3.10 (0.71)	2.83 (0.59)
Stereotype-unrelated trait	3.78 (0.80)	4.30 (0.88)	3.58 (0.81)	3.03 (0.70)

Numbers outside of the parentheses represent the means, while numbers inside of the parentheses represent the standard deviations.

**Table 3:** Perceived dispersion of stereotypic and stereotype-unrelated dimensions across the priming and variability conditions in Experiment 1.

Type of prime	Stereotypic dimension		Stereotype-unrelated dimension	
	Heterogeneous condition	Homogeneous condition	Heterogeneous condition	Homogeneous condition
Counter-stereotypic trait	2.38 (0.74)	1.58 (0.59)	1.78 (0.62)	1.65 (0.58)
Stereotype-unrelated trait	1.83 (0.71)	1.53 (0.55)	2.25 (0.67)	1.60 (0.59)

Numbers outside of the parentheses represent the means, while numbers inside of the parentheses represent the standard deviations.

### Suspicion

As fewer than 2% of participants (n = 3) made speculations linking the priming and group impression tasks, and these speculations were tangential to the true purpose of the experiment, no responses were omitted from analysis.

### Trait descriptiveness

Values on the stereotypic rating scale were inverted, ensuring that higher values (ranging from 1 to 9) corresponded to more stereotypic views of the target group. Two 2-way analysis of variance tests (ANOVA) (2 prime: counter-stereotypic or stereotype-unrelated × 2 variability scenarios: homogeneous or heterogeneous) were computed, one for ratings on the stereotypic dimensions (un/intelligence for construction workers; un/friendliness for bouncers) and one for ratings on the stereotype-unrelated dimensions (un/intelligence for bouncers; un/friendliness for construction workers). Corrections for multiple comparisons performed through the 2-way ANOVAs for the stereotypic and the stereotype-unrelated dimensions, beyond the follow-up planned comparison, were applied using the Holm-Šídák test to calculate the adjusted *p* values, as reported below.

Ratings for stereotypic dimensions (Table 1) revealed two significant main effects. In a heterogeneous scenario, stereotypic dimensions were rated as less descriptive of the group (*M* = 6.38, *SD* = 1.04), than in a homogeneous scenario (*M* = 8.19, *SD* = 0.83), F(1, 156) = 145.46, *p* = 0.008, *MSE* = 0.903, η_p_^2^ = 0.48. Additionally, mean evaluations were significantly higher when the prime was stereotype-unrelated (*M* = 7.79, *SD* = 0.88), than when it was counter-stereotypic (*M* = 6.78, *SD* = 1.00), F(1, 156) = 45.39, *p* = 0.008, *MSE* = 0.903, η_p_^2^ = 0.23. A statistically significant two-way interaction was also found, (F(1, 156) = 43.18, *p* = 0.008, *MSE* = 0.903, η_p_^2^ = 0.22), and a planned contrast indicated that the effect of decreased descriptiveness of stereotypic dimension was significantly stronger for the counter-stereotypic prime in the heterogeneous condition (M = 5.38) than for the other three conditions joined together (M = 7.92), *t*(156) = 76.60, *p* = 0.008, *d* = 0.04.

Ratings on the stereotype-unrelated dimensions (Table 1) revealed two significant main effects. There was a main effect of the perceived variability manipulation, F(1, 156) = 31.12, *p* = 0.008, *MSE* = 1.191, η_p_^2^ = 0.17, indicating increased descriptiveness of stereotype-unrelated dimensions in the heterogeneous scenario (*M* = 5.74, *SD* = 1.15; than in the homogeneous scenario, *M* = 4.78, *SD* = 1.00). The second main effect was for the type of prime (M_counter-stereotypic prime_ = 5.06, M_stereotype-unrelated prime_ = 5.45, F(1,156) = 5.04, *p* = 0.026, *MSE* = 1.191, η_p_^2^ = 0.03), suggesting stereotype-unrelated primes significantly increased how characteristic participants thought stereotype-unrelated dimensions were for the group. However, a statistically significant two-way interaction between perceived variability manipulation and type of prime qualified these main effects, F(1, 156) = 6.43, *p* = 0.024, *MSE* = 1.191, η_p_^2^ = 0.04. A planned contrast showed that the effect of increased descriptiveness of stereotype-unrelated dimensions was significantly more pronounced when the prime was stereotype-unrelated, in the heterogeneous condition (M = 6.15) than for the other three conditions joined together (M = 4.96), *t*(156) = 55.75, *p* = 0.008, *d* = 0.01.

#### Distribution choice measures of centrality and variability

Since the distribution matrix from which participants made their selection independently manipulated central tendency and dispersion, the measure is relatively resistant to the artifactual consequences of central tendency polarization in dispersion parameters ^23,67^. The subsequent analyses concentrate independently on each of these parameters. Again, corrections for multiple comparisons performed through the 2-way ANOVAs for the stereotypic and the stereotype-unrelated dimensions were applied using the Holm-Šídák test to calculate the adjusted *p* values, as reported below.

Perceived central tendency parameter

We conducted two double-factors ANOVAs (2 prime: counter-stereotypic or stereotype-unrelated × 2 variability scenario: homogeneous or heterogeneous) to analyze distribution choices on stereotypic and on stereotype-unrelated dimensions. Higher values on the stereotypic dimension measure (ranging from 1 to 5) indicate more stereotypical views of the target group. The results closely mirrored those of trait descriptiveness measure, given their shared sensitivity to the central tendency (Table 2). A main effect of perceived variability manipulation was observed, F(1, 156) = 71.61, *p* = 0.008, *MSE* = 0.788, η_p_^2^ = 0.32, with mean evaluations less stereotypic in a heterogenous scenario (*M* = 3.06, *SD* = 0.91) than in a homogeneous scenario (*M* = 4.25, *SD* = 0.85). Additionally, a main effect of the type of prime emerged, F(1, 156) = 29.53, *p* = 0.008, *MSE* = 0.788, η_p_^2^ = 0.16, showing that stereotype-unrelated primes led to more stereotypic evaluations (*M* = 4.04, *SD* = 0.84) than counter-stereotypic primes (*M* = 3.28, *SD* = 0.93). A statistically significant two-way interaction was also revealed, F(1, 156) = 22.29, *p* = 0.008, *MSE* = 0.788, η_p_^2^ = 0.13. A planned contrast indicated that the decrease in descriptiveness of stereotypic dimensions, when the prime is counter-stereotypic in a heterogeneous scenario, was significantly stronger (M = 2.35) compared to the other three conditions joined together (M = 4.09), *t*(156) = 39.76, *p* = 0.008, *d* = 0.04.

Regarding the stereotype-unrelated dimensions, two significant main effects were observed (Table 2). A main effect of perceived variability manipulation, F(1, 156) = 13.59, *p* = 0.008, *MSE* = 0.501, η_p_^2^ = 0.08, indicated higher mean evaluations on stereotype-unrelated dimensions in a heterogenous scenario (*M* = 3.34, *SD* = 0.76), than in a homogeneous scenario (*M* = 2.93, *SD* = 0.65). There was also a main effect of type of prime (M_counter-stereotypic prime_ = 2.96, M_stereotype-unrelated prime_ = 3.30, F(1,156) = 9.10, *p* = 0.008, *MSE* = 0.501, η_p_^2^ = 0.06), with stereotype-unrelated prime significantly increasing how characteristic participants perceived stereotype-unrelated dimensions to be. No significant two-way interaction was found, F(1, 156) = 1.51, *p* = 0.221, *MSE* = 0.501, η_p_^2^ = 0.01. A planned contrast, aligned with the pattern from the trait descriptiveness measure emerged, showing increased descriptiveness of stereotype-unrelated dimensions when the prime was stereotype-unrelated in a variability condition was significantly stronger (M = 3.58) than the remained conditions together (M = 2.98), *t*(156) = 50.76, *p* = 0.008, *d* = 0.01.

### Perceived dispersion parameter

Next, we conducted two 2-way ANOVAs (2 prime: counter-stereotypic or stereotype-unrelated × 2 variability scenario: homogeneous or heterogeneous) to analyze distribution choices on stereotypic and stereotype-unrelated dimensions. Higher values (ranging from 1 to 3) on this measure indicate the group is perceived as more variable (see Table 3). Results revealed two significant main effects. First, the perceived variability manipulation showed a significant effect, F(1,156) = 28.22, *p* = 0.008, *MSE* = 0.429, η_p_^2^ = 0.15), suggesting that the heterogeneous condition received higher perceived dispersion along the stereotypic dimension (*M* = 2.10, *SD* = 0.73), than the homogeneous condition (*M* = 1.55, *SD* = 0.57). Second, a main effect of type of prime was observed, F(1, 156) = 8.40, *p* = 0.001, *MSE* = 0.429, η_p_^2^ = 0.05, with counter-stereotypic primes leading to higher perceived dispersion (*M* = 1.98, *SD* = 0.67) than stereotype-unrelated primes (*M* = 1.68, *SD* = 0.63). A statistically significant two-way interaction was also found, F(1, 156) = 5.83, *p* = 0.034, *MSE* = 0.429, η_p_^2^ = 0.04. A planned contrast supported the hypothesis that counter-stereotypic priming, in the heterogeneity scenario, more strongly increased perceptions of dispersion in the target group, along the stereotypic dimensions (M = 2.38), when compared to the three remained conditions together (M = 1.64), *t*(156) = 33.60, *p* = 0.008, *d* = 0.02).

The analysis for the stereotype-unrelated dimension also indicated a noteworthy significant effect of perceived variability manipulation on distribution choices F(1,156) = 15.83, *p* = 0.008, *MSE* = 0.379, η_p_^2^ = 0.09. Participants exposed to a heterogeneous report perceived the group as more dispersed in the stereotype-unrelated dimensions (*M* = 2.01, *SD* = 0.65), compared to those in the homogeneous condition (*M* = 1.63, *SD* = 0.59). Additionally, there was a main effect of the priming condition F(1,156) = 4.76, *p* = 0.034, *MSE* = 0.379, η_p_^2^ = 0.03, indicating that stereotype-unrelated primes increased the participants’ perception of the group as more dispersed on that dimension (M_counter-stereotypic prime_ = 1.71, M_stereotype-unrelated prime_ = 1.93) (see Table 3). Furthermore, a significant two-way interaction was observed, F(1, 156) = 7.27, *p* = 0.024, *MSE* = 0.379, η_p_^2^ = 0.05, highlighting that stereotype-unrelated priming, in the heterogeneous condition, had a significantly more pronounced effect in increasing perceptions of dispersion (M = 2.25), when compared to the other three joined conditions (M = 1.68), *t*(156) = 34.91, *p* = 0.008, *d* = 0.01) (see Table 3).

When participants were presented with a more intricate portrayal of professional groups that emphasized the plausible dissimilarities among members, they exhibited a greater tendency to integrate contextually primed counter-stereotypic traits into the subsequent stereotype reported for a specific professional group. This was evident in the typicality ratings and perceived central tendency measures. The target groups, when exposed to such a context, were also perceived as more variable which was reflected in the preference for flatter distributions. There was a reduction in members with stereotypic attributes and an increase in members with counter-stereotypic attributes, aligning with findings from prior research, using a similar paradigm ^28^. In the homogeneous scenario, where group members were perceived as uniform, there appeared to be a limitation on the adaptability of stereotypes to contextual nuances. Both information conflicting with prior beliefs and stereotype-unrelated information struggled to integrate into the stereotype.

These findings highlight that directing attention to the diversity within professional group members enhances the likelihood of incorporating both counter-stereotypic and stereotype-unrelated information primed into the stereotype of a specific professional group. Moreover, the observed pattern of results implies that the underlying mechanism to this effect involves a modification in participants’ overall perception of variability within professional groups members. Information portraying members as dissimilar diminishes the inclination to associate characteristics in an all-or-nothing manner, altering group’s stereotypical picture. Conversely, perceiving members as highly similar seems to impede the acceptance of counter-stereotypic and stereotype-unrelated constructs into the reported stereotype, preserving the group’s stereotypical characterization. However, the pivotal role played by perceived group variability seems to be triggered by the presence of an activated primed construct coinciding with the nature of the dimension measured.

## Discussion

The primary objective of the current research was to empirically validate the proposition that altering individuals’ overall perceptions of variability among members of professional groups would lead to the integration of contextually primed counter-stereotypic information into the stereotype content of a targeted professional group, resulting in a transformation of the stereotype. This was accomplished by directly manipulating perceived group variability through two pretested scientific-communication reports designed to enhance overall perceived similarity or dissimilarity within-group members sharing the same professional group.

The main finding indicated that heightening the plausibility of group members as being dissimilar increased the likelihood of incorporating counter-stereotypic information activated into the stereotype representation of a targeted professional group, leading to a less stereotypic description exhibited by the patterns obtained with typicality ratings and central tendency measures in the stereotypic dimension. A similar pattern was observed for the stereotype-unrelated dimensions when a stereotype-unrelated prime was used. Additionally, perceiving members as dissimilar increased perceptions of variability in both stereotypic and stereotype-unrelated dimensions. Therefore, stereotype generalization occurred not only through changes in perceived variability measures ^28^, but also, in perceived central tendency of the group as a whole.

Importantly, the findings suggest that merely increasing perceived group variability in anticipation was not enough to produce stereotype changes in the stereotypic dimension, unless accompanied by the counter-stereotypic prime. It appears that typicality and central tendency ratings in the stereotypic dimension remained unchallenged in the condition that a stereotype-unrelated prime was used. These results imply the stereotype was not necessarily weakened by the heterogeneity condition alone, but rather that individuals may become more receptive to atypical information in its presence. Thus, counter-stereotypic trait priming seems to be a necessary step to change estimations of the likelihood that two opposed traits occur in the group as a whole, with the stereotype becoming more easily changed by its meaningful central tendency.

Our proposition is that maintaining an all-or-nothing relationship between a stereotypic trait and a social group becomes challenging when participants are primed to perceive members of the same groups as heterogeneous and, subsequently, face activated counter-stereotypic information. If members are seen as dissimilar, it becomes nearly impossible to attribute a characteristic to all of them as if they were interchangeable, particularly because the presence of a counter-stereotypic information calls attention to that diversity. This suggests that the concept of group membership may lose some of its resistance to change in a heterogeneous group, implying that some members possess certain characteristics to a lesser extent than others, in that typical and atypical members might even be equally common, and the appearance of an atypical individual is not at all surprising. Therefore, facing counter-stereotypic information in a heterogeneous context may have brought participants’ attention to how attributes may vary in within-group members, revising the central tendency as the perceived variability mental representations of the group. From this, we can potentially increase perceivers’ attributions of traits that are counter-stereotypic to the group by, in advance, augmenting the perceived variability of groups.

Some authors ^22,35^ argue group membership can strictly serve as a guide for behaviors only if the group is homogeneous; once the group becomes heterogeneous, group membership is no longer a diagnostic cue for behaviors. Thus, elevating the salience of heterogeneity among members may render group membership less diagnostic for stereotypic traits, (see ^22,35^). High central tendency and low variability stereotypes are highly useful due to their diagnostic nature, providing perceivers with specific and predictive information ^35^. A similar reasoning was framed by ^68^, when addressing the ‘decategorization’ type of generalization, which refers to change in the perceived usefulness (or meaningfulness) of a social category for identifying and classifying new individuals. Our results stress that, unless perceived heterogeneity coincides with the contextual activation of a counter-stereotypic construct, the exposure to such perceived variability framework won’t be considered, and applied to the subsequent assembling of the target group stereotype, preventing the extension of the effects to the discernible revision in central tendency parameters of its stereotype ^60,61^.

Assuming that stereotypes are a mental instrument of differentiation between groups, when stereotypic dimensions lose their status, does that mean stereotypic traits are no longer indicative of the social group and able to uphold group identity in the social context? We revisit the contention suggested by ^44^ to clarify this point. We argue it fosters a heightened awareness and appreciation for the diversity among group members regarding those characteristics, thereby rendering it easier to tailor responses to the prevailing context. However, this does not inherently signify the stereotype has disappeared.

An interesting constraint to the observed effect involving the counter-stereotypic information worth exploring is the nature of the counter-stereotypic information primed—especially when the corresponding stereotypic dimension is expected to lack variability among members of a professional group (i.e., members are all alike). Mental representations of social groups likely differ depending on whether a stereotypic dimension encountered is expected to be more or less variable. Such attributes may be so central to a group that eliminating or reducing their incidence would fundamentally alter our understanding of the group.

Sloman and colleagues argued the most central features are the most immutable ^69,70^. These authors mention as an example, the roundness of oranges. The notion of oranges would not change if all oranges were round, and we imagined one that was not ^69,70^. However, if a wheel lacks roundness, it necessitates a complete reconception to maintain its mental classification as a wheel ^69,70^. In a similar vein, dockworkers are not perceived as dockworkers unless resistant and strong. Thus, what would we expect if fragility was the counter-stereotypic trait contextually primed, when participants have been previously exposed to a perceived variability scenario? Our best prediction is that fragility would not be accepted as representative of one's beliefs about dockworkers. Fragility would likely not be incorporated into the central tendency and not be reflected in measures of perceived dispersion of members over corresponding stereotypic dimensions.

Despite the potential theoretical advancements this study provides, there are nonetheless a few limitations worth noting. Perhaps most importantly, the target groups tested in the present study were limited in number which admittedly challenges the overall strength and generalizability of the findings, highlighting the need for the replication of the results to account for more professional groups. Furthermore, present research did not investigate the persistence of stereotype reduction effects observed. Forthcoming research could explore this inquiry, by implementing a follow-up procedure to assess whether these effects endure overtime or are merely transient.

Finally, most researchers in the stereotype domain primarily focus on the biasing effects of stereotypes on impressions and judgments ^1,2^. These biases are often associated with the stereotypic traits linked to social categories and frequently accessible in social encounters ^56–58^. The present research underscores perceived variability may be a crucial determinant of the extent to which stereotypic information may be readily assumed to shape assessed beliefs and applications.

## Data Availability

The datasets generated during the current study are not publicly available, but are available from the corresponding author on reasonable request.
